# A Case of Placenta Prævia

**Published:** 1866-11

**Authors:** John Reid

**Affiliations:** Chicago


*A Case of Placenta Prævia.* By JOHN REID, M. D., Chicago.

On the 2d of February last, I was called to see Mrs. P——, who was in the seventh month of her third pregnancy. She suffered from indigestion, constipation and general debility; but her principal complaint was of a steady, persistent pain in the right hypochondriac region which had troubled her for several months. There was no special tenderness of the part on pressure. The persistence of the pain, rather than its severity, occasioned her great anxiety; and she expressed her fears that there was something wrong in the position of the fœtus, or in the state of the womb, which would compromise her safety when she came to be delivered. I could not form a decided opinion as to its cause, but was inclined to think it might be dependent on the disordered state of her digestive organs. Under treatment directed to the improvement of that condition, she materially improved in health and spirits, but the pain continued.

I did not see her again until the morning of March 26th, when she sent for me in urgent haste. She had been seized a few hours before—while lying quietly in bed—with flooding to an alarming extent, which still continued, though in much less degree. Vague pains in the back and abdomen had preceded and accompanied it. She was just entering upon her ninth month. I at once administered half a drachm of fluid extract of ergot, and prescribed smaller doses every hour during the continuance of the flooding. I remained about two hours, when the hemorrhage had almost ceased. The discharge ceased altogether soon after I left in the morning, and her pains also subsided. No pains appeared to have been induced by the ergot.

27th.—Patient passed a restless night. Complained of acute headache, ringing sound in her ears, strong pulsation in her temples, and an uncontrollable state of alarm. Her countenance was flushed, pulse full and bounding, but soft and compressible. No return of hemorrhage. Prescribed cold applications to head, and an ounce of castor oil, as her bowels had not been moved for several days. In the evening she was more comfortable; bowels had freely moved. She had not slept and was dreading another wakeful night. Prescribed a third of a grain of sulphate of morphia.

28th.—Passed a comfortable night, and feels better in all respects. Enjoined rest in bed and careful avoidance of excitement.

A few days afterwards, I called, as I had not heard from her, and found her up and feeling comparatively comfortable, but very weak. I insisted on her going to bed and remaining there, which she did, and I heard no more of her until the morning of the 7th of April, when I was again summoned to her assistance. A profuse gush of blood had taken place about 3 o'clock A. M., while lying in bed.

The discharge had, as in the former instance, been preceded and accompanied by slight pains. By the time I arrived, which was 7.30 A. M., the flooding had greatly abated. She had commenced the use of the ergot mixture (℞ Vin. ergotæ, ℥iiss., fl. ext. ergot. ℥ss., M., dose ʒj.) on the first occurrence of flooding, and kept it up every hour as directed. I remained an hour—continued the use of the ergot, and had the satisfaction of finding the hemorrhage rapidly abating. In the course of the day it ceased entirely, and the pains which had been quite irregular paased off. For the first time, I made an examination in hope of finding an explanation of the hemorrhages. The os was closed, the cervix very short, but not quite obliterated. On pressure with the finger the walls of the uterus seemed soft and cushion-like. The sensation conveyed by the touch confirmed the conclusion I had previously reached, that the case was one of placenta prævia.

During the interval of these attacks my patient had rallied well from the effects of the first. Rest in bed, with judicious diet and nursing, had done much to restore the loss, so that, although she was much prostrated by it, she bore the second attack better than I could have expected.

8th.—Under the influence of opium she had passed a quiet night and continued comfortable through the day. She partook freely of beef-tea and milk, which agreed well with her.

17th.—Called in haste at 9 A. M., in consequence of another attack of hemorrhage. She had experienced slight pains during the preceding day and evening, which increased during the night, recurring at regular intervals. A slight flow had taken place in the night, but not sufficient to excite much alarm until about 6.30 A.M., when it became profuse. When I arrived my patient's blanched appearance and feeble pulse indicated the necessity for prompt interference to arrest the hemorrhage. She had now arrived at her full term, and the regular recurrence of pains showed unmistakably that labor had commenced.

The time for decisive action had arrived. What that action should be could only be determined by the condition of the os uteri. Providing myself with a knitting needle, in case I should conclude to puncture the membranes, I made an examination, and found the os closed and apparently rigid. On attempting to introduce the finger, I found the posterior lip yielded, so that I passed my finger and felt the placenta implanted over the os. It was firmly attached over the anterior lip, but posteriorily it was partially separated, and I could introduce my finger for a short distance in that direction. Introducing my whole hand into the vagina, as the os uteri was high in the pelvis, I pushed my finger onwards, separating the placenta in search of its edge, beyond which I intended to tap the bag of waters. I could not reach it,—the presentation of the placenta being at least nearly central.

The contracted state of the os interposed an insuperable obstacle to the introduction of the hand for the purpose of effecting delivery, and equally opposed the accomplishment of Dr. Simpson's plan of removal of the placenta. Had Dr. Barnes' dilator been accessible to me, I should have used it. As it was, three practicable methods, or a combination of them, remained to me: 1st, Rupture of the membranes through the substance of the placenta; 2d, Dr. Barnes' method of partial separation of the placenta; 3d, The tampon. The first I decided against on account of the risk to the child.

I determined on the second, and then if flooding should continue, to use the tampon. Accordingly, I separated the placenta as far as my finger could reach over the posterior lip, but on attempting to sweep it around over the anterior, I found it impossible to do so, owing to the firmness of its attachment. I then removed my hand, and found to my surprise that the hemorrhage had ceased. I say to my surprise, for I supposed my failure to separate the placenta from the entire lower zone of the uterus would necessarily entail the penalty of continued and increasing flow.

It was now 8.30 A. M., and during all the time which had elapsed since the commencement of discharge in the night, my patient had been taking the ergot mixture every hour as prescribed, and in addition, I had administered a drachm of the fluid extract immediately on my arrival. On the cessation of the hemorrhage, the ergot was discontinued. The pains continued regular but feeble, and notwithstanding the quantity she had taken, had nothing of the ergotic character. On the contrary, as the day advanced they gradually declined, so that from 10 A. M. until about 4 P. M. hardly any were felt. I remained with her, except at brief intervals, throughout the day. Towards evening the pains returned with more frequency, and with them a renewal of the discharge. An examination at this time showed but little change in the condition of the os. The anterior lip remained rigid as before, while the posterior was more dilatable. I recommenced the use of the ergot mixture, and by 7 P. M., persistent pains, with little intermission, showed the decided action of the drug; a slight discharge occurring at intervals. I now discontinued the ergot and sat down to wait results. By 8 o'clock the pains had died away,—the os was more dilatable, but not dilated, and scarcely a sign of discharge.

8.30 P. M.—Slight increase of discharge, but no pains. Called Dr. Paoli in consultation. On ascertaining the condition of the os, he urged the use of gallic acid, 15 grs., and tartar emetic, $\frac{1}{4}$ gr., every half hour, four doses of which were given.

11 P. M.—Still no pains; os unchanged; slight continuous discharge. Patient by this time was fast losing strength and courage, and it was evident she must succumb unless delivery could be speedily effected. No warrantable degree of force would penetrate that rigid os uteri. What, then, was to be done? I was opposed to the use of the tampon, both on account of the passive state of the womb and the extensive separation of the placenta, which would permit the efflux of blood between the membranes and uterine walls. There seemed to be no resource but to administer stimulants and wait for such a degree of dilatation as was necessary to effect delivery.

12.30 A. M.—Os uteri much more relaxed and dilatable, (but only in its posterior lip;) discharge increasing. At my request, Dr. Paoli now proceeded to make an attempt to deliver through an opening made in the placenta.

After a tedious effort, he succeeded in making an opening large enough to introduce the hand, and then requested me to relieve him, as he was too much fatigued to proceed. Before his hand was withdrawn I administered a drachm of fluid extract of ergot, and immediately as he withdrew his hand introduced my own, and pushing it on quickly through the placenta, found the head presenting, occiput backwards. Pushing onwards I found a knee, behind which I hooked my finger, and turned without much difficulty. It required considerable time and force, however, to bring the child, which was large, through the comparatively unyielding os; but we concluded that prompt delivery was necessary to the safety of the mother, while it afforded us the only hope of saving the infant. As it was, the delay in the passage of the head was unavoidably so great that I had given up all hope of the child, when I felt it make a convulsive movement. Stimulated by this sign of life, I renewed my efforts, and soon succeeded in effecting its delivery. To all appearance it was dead. With Dr. Paoli's assistance, a combination of Sylvester's and Marshall Hall's ready method was diligently practiced, with alternate dippings in hot and cold water. After, perhaps, five minutes, a feeble gasp encouraged us to proceed. By and by came another and another at considerable intervals, until at length, after fully half an hour from the time of delivery, a feeble wail and the occurrence of regular respiration rewarded our efforts.

In the meantime, having satisfied ourselves that on the part of the mother the flooding had ceased, and the womb had well contracted, she was entrusted to the care of a nurse, who was instructed to grasp the uterus through the parietes and exert steady pressure thereon.

The safety of the infant being now insured, I requested Dr. Paoli to see to the mother. The placenta had not come away, and on introducing his hand to remove it, found it was impossible entirely to detach it, owing to its close adhesion over the anterior aspect of the womb. Though fearfully exhausted, the condition of the patient was promising. A full dose of opium served to tranquilize her nerves and steady her pulse. I remained until daybreak, when I left with good hopes of both mother and child. The child lived and performed all its functions well for a week, when it was seized with symptoms of intussusception and died.

The mother remained under treatment for some time, but progressed favorably from day to day. The small retained portion of placenta came away a few days after delivery.

Owing, I suppose, to the great loss of blood, no lacteal secretion was formed. To-day, five months from her delivery, she is in good health, although she has not regained her former vigor.

